# Looking Forward

**Published:** 1890-01

**Authors:** S. H. Preston


### LOOKING FORWARD.

BY S. H. PRESTON.

No. 5.

The sole aim of these articles is to show that population is slowly but surely outstripping the means of subsistence, and that pauperism is outstripping population. This Dr. Babbitt does not directly dispute. But to him the calamitous consequences appear too far off in the future for present apprehension. He believes that before the day of dire distress Bellamy's ideal Boston will be in full blast and Pacific Paradise completely planted.

The Doctor declares there will be no danger "if we can only bring about a righteous division and distribution of the results of labor."

Very true. If we could only establish the kingdom of heaven on earth there would be no more evil, nor naughtiness, nor unhappiness.

A righteous distribution of food would have prevented all the famines in the past. But the famines are fearful facts, all the same. When we were sending shiploads of supplies to suffering Ireland, Ireland was exporting food enough to have saved all the starvation. So with China. At the time nine and a half millions were perishing from hunger ere was plenty of food in the empire for the entire population. But it could not be distributed. The starvation was in one section, the supplies in another. All the efforts of the Chinese government to convey relief to the destitute districts were frustrated because of bad roads and insufficient carrying facilities, and because the famine-frenzied folks along the lines of transportation killed and devoured the pack-horses and mules in their distress.

Grant that corn enough can be grown in Kansas to feed a kingdom. But the corn must be carried from Kansas to the kingdom to do any good. If the hungry people have not the means to purchase or procure the corn, they will perish unless some other kingdom kindly comes to their rescue. The Kansas farmers will use their corn for fuel, as they did a few years ago, finding it cheaper than fire wood, if there be no monied market for it.

To-day there is prospective starvation of farmers in the Dakotas while their grain bins are bursting with an abundant stock of wheat. These wheat-growers must get their crops to Minneapolis to be ground and get the flour back, and they have not the means of paying for the milling and transportation. They have wheat, but not money. Flour is cheap in Dakota, but there is no money to buy it.

Of course the causes for this condition of affairs are local, and arise chiefly from the cupidity of man and the competitive system of business. But it is this very cupidity and competition which will baffle the Bellamys and Babbitts in bringing about the "righteous division and distribution" they desire. It is feared many folks will die of famine before reformers root out avarice from the human heart and establish on earth the reign of righteousness.

Dr. Babbitt betrays a remnant of the old notion that the Providence that sends mouths also sends meat for them. But it may be noted that where the mouths are put in one place and the meat in another, as was the case in China, millions will starve for aught Providence does to make the proper connection between them.

Dr. Babbitt has an idea that the want in the world is due to a wrong adjustment between capital and labor. The question is wholly one of wealth and want. Now, what is wealth ? Surely not gold. For, taking that as a criterion, the world is rapidly declining in wealth. In 1852 the annual production of this precious metal was $182,500,000. In spite of the enlarging demand for it, due to increase of population, commercial enterprise and expensive luxuries, the world's annual gold production now falls short of $90,000,000. As no new mines are discovered, and old ones are being exhausted, requiring extra labor to extract the metal from the ore, while labor is diverted to other industries more and more each year, the decline is likely to continue faster than formerly.

The soil is the sole source of wealth. Real riches are agricultural productions. The farmers and their farms are the foundations of a people's permanent prosperity. Manufactories are carried on by capital, and render increase of capital in return ; but all the capital in the world will fail to fill a want for food, and land alone is the basis of food. The mechanic at the forge only hammers matter into other forms ; the weaver at the loom only fashions fabrics into other figures. They only change the conditions of the material upon which they work, but create nothing. But the cultivator of the soil calls into existence productions that did not previously exist, and is therefore a creator.

Were there but one kernel of corn in the world, it would be worth more than the Kohinoor ; were there but one potato, it would be priceless. The farmer would plant them in his furrowed field. Then nature would consummate a new creation, would work a miracle of multiplication. The rain and the dew would descend ; the sun would shine, and down in the genial ground the seeds would germinate and grow into many. The farmer would gather the crop and use it to seed the productive soil again. By and by he would get crops by bushels. He plows and plants his prolific acres, and for every bushel buried reaps forty in return. The bushels breed bountifully down in the bosom of mother earth. Folks come and give him gold for what he grows. What has he done ? Simply converted sunshine and soil into gold with the aid of that mysterious alchemist, nature. His corn is worth more than crown jewels in a starving kingdom. He has produced wealth where there was none before. It is wealth that is evoked ; wealth that grows and multiplies and gives life.

Suppose a country cut off from all communication with the rest of the world. It is inhabited by an equal number of farmers and manufacturers. The former furnish the food, the latter operate the furnaces and factories. The two classes increase alike. Finally the farmers find that they cannot make their food products keep pace with population. Which will suffer first, the agriculturists or the artisans ? The former have only fed the latter from their abundance, and, of course, cannot continue to do so further than they have supplies to spare. So some of the artisans must starve or turn agriculturists and go to growing their own food. As they increase in number, more and more of them will be forced to forsake the forge for the farm. At last it will require the utmost labor of all to get a bare living from the land. The artisans will abandon their shops ; the spindles will stop ; the furnace fires go out ; the factories fall to ruins, and at last the farmers will fail to raise sufficient food for their own increasing numbers.

Suppose one country is wholly peopled by wool-growers and weavers, another by gold-miners, and another by wheat-growers. With proper commercial exchange, all might prosper and multiply in population. The miners could maintain themselves as long as they could get woolens and wheat with their gold ; the weavers would fare well as long as they could get wheat with the gold paid for their goods, or in exchange for them. Were the gold to give out, the miners would be compelled to change their country and occupation, and choose between wool-working and wheat-growing. They would be no worse off for means of living. But were the wheat crop to fail the wool folks would have to kill their sheep for food and go to growing wheat for themselves or starve.

Suppose England had been shut in by the sea a century ago and had been obliged to rely upon her own scanty area of soil for supporting her people. What would have been the fate of her vast populations poured out by emigration ? How many foundries and factories would there be in Manchester and Birmingham to-day ?

But the steam carrying and commercial facilities of the times are rapidly making mankind one family, transporting the impoverished to places of plenty, and supplying one nation's needs from another's abundance through the channels of profitable trade. A manufacturing people can thus be fed by an agricultural people, and former wide-spread famines be averted. As long as a handicraft country can buy breadstuffs from a farming one, or the population of an impoverished country can escape to a prosperous one, the calamities of overcrowding may be postponed. But the evil end will come eventually, as food-producing land is a fixed quantity and even the earth is limited, while the power of human multiplication, as maintained by Mill and Darwin, may be regarded as infinite.

"When earth produces free and fair
The golden waving corn ;
When fragrant fruits perfume the air,
And fleecy flocks are shorn ;
Whilst thousands move with aching head,
And sing the ceaseless song,
'We starve, we die, oh, give us bread !'
There must be something wrong."

There is a great deal that is wrong in all the relations of life. But the overwhelming wrong of the world is the reckless and unrestricted reproduction of the race, the breeding of beings the world does not want and cannot comfortably care for.

